# Parent Satisfaction With Pediatric Anesthesia—A Cross‐Sectional, Multicenter Study in Norway

**DOI:** 10.1111/pan.15094

**Published:** 2025-03-15

**Authors:** Ann‐Chatrin Linqvist Leonardsen, Berit Taraldsen Valeberg, Annette Danielsen, Cecilia Jonth, Ingrid Marie Brunborg, Endre Dingstad Jørgensen

**Affiliations:** ^1^ Østfold University College, Østfold Hospital Trust Halden Norway; ^2^ Oslo Metropolitan University Oslo Norway; ^3^ University of Southeastern Norway Oslo Norway; ^4^ Department of Anesthesia Ringerike Hospital, Vestre Viken Hospital Trust Ringerike Norway; ^5^ Department of Anesthesia Intensive Care and Operating Theatre Services, Bærum Hospital, Vestre Viken Hospital Trust Sandvika Norway; ^6^ Vestfold Hospital Trust Tønsberg Norway

**Keywords:** information, parent satisfaction, pediatric anesthesia, postoperative symptoms, questionnaire

## Abstract

**Background:**

Parental satisfaction with anesthetic care is utilized as a proxy for child satisfaction. The evidence base regarding parent satisfaction with pediatric anesthesia care is limited. The aim of the current study was to assess (1) parent satisfaction with pediatric anesthesia in three hospitals, (2) potential differences across hospitals, and (3) potential associations between parent satisfaction and the child's age, surgical specialty, hospital, whether the parent previously had followed a child to surgery, and relation to the patient.

**Methods:**

The Norwegian version of the “pediatric anesthesia parent satisfaction survey” questionnaire (NPAPS) was used. Descriptive statistics, Kruskal–Wallis test and linear regression analysis were used to analyze the data.

**Results:**

In total, 234 parents responded. Most respondents agreed that their child had received the highest quality care during the surgical experience (96.4%–97.4%). Nearly all respondents agreed that their questions were responded to (94.6%–97.6%), information was understandable (96.4%–97.4%), the amount of information was appropriate (96.4%–97.4%), the child's integrity was respected (94.6%–97.4%), the child was treated respectfully and professionally (96.45%–97.6%), and that personnel paid attention to the parent's concerns (93.8%–94.7%). Respondents disagreed with being explained how the child might feel physically and emotionally after anesthesia (9.5%–10.5%) and also disagreed with being satisfied with the way the child fell asleep and woke up from anesthesia (5.3%–7.1%). No factors were associated with the level of parent satisfaction, and there were few differences between hospitals.

**Conclusion:**

Even if parents are overall satisfied with anesthesia care, improvement areas regarding the preparation of parents and control of postoperative symptoms were detected.

## 1 Background

In high‐income countries, about one in seven children has had at least one anesthetic procedure before the age of 4 years [1]. Anesthetic services are conducted in high‐pressure and high‐technological environments that are unfamiliar to parents and their children [2]. Also, the anesthesia management of pediatric patients for surgical or diagnostic procedures poses a relatively high risk of critical events, and there is a large variability in the practice of pediatric anesthesia throughout Europe [3]. Studies have shown that adverse experiences in pediatric patients can negatively affect medical outcomes, leading to illness, discomfort, pain, and distress [4]. Additionally, pediatric patients show a range of distress behaviors in response to medical procedures and anesthesia, ranging from verbalizations of fear or protests to escaping attempts [5]. Consequently, pediatric surgery involves a considerable amount of stress for pediatric patients and their parents [4, 6].

### 1.1 Summary of Existing Literature

Patient satisfaction is frequently used to measure healthcare quality [7]. Studies indicate that children's experiences are linked to and can be affected by their parents' experiences [4]. Many pediatric patients are unable to evaluate and express their experiences, and parental satisfaction with anesthetic care is utilized as a proxy for child satisfaction [8]. A 2013 systematic review of satisfaction measures in anesthesia found 34 psychometrically evaluated instruments. Of these, only six were designed for pediatric patients [9]. In 2021, Chua et al. [10] identified 14 unique satisfaction measures of perioperative pediatric care. All of them relied on responses from parents. Six dimensions of satisfaction with anesthetic pediatric care were identified: “information giving,” “involvement in decision‐making,” “hospital experience,” “staff report and communication,” “quality of anesthesia or nursing care,” and “postoperative symptom control.” Few studies have been conducted in Western countries focusing on parent satisfaction with pediatric anesthesia care.

In Norway, nurse anesthetists and anesthesiologists, as well as operating room nurses and surgeons, comprise the team around pediatric patients and their parents. Most commonly, either the nurse anesthetist or the anesthesiologist performs the pre‐anesthetic assessment [11], either some time before surgery or the same day. In many hospitals, the patients are admitted to a pediatric or a preoperative day‐case ward the day of surgery or procedure. Here, nurses are responsible for preparing patients and parents before transferring them to the operating room. To our knowledge, no studies have explored parent satisfaction in a Norwegian perioperative setting. Hence, the aim of the current study was to assess (1) parent satisfaction with pediatric anesthesia in three hospitals in Southeastern Norway, (2) potential differences in parent satisfaction across hospitals, and (3) potential associations between parent satisfaction and factors such as the child's age, surgical specialty, hospital, whether the parent previously had followed a child to surgery, and relation to the patient.

## 2 Materials and Methods

The study had a quantitative, multicenter design, using a questionnaire. The study adheres to the STrengthening the Reporting of OBservational studies in Epidemiology (STROBE) guidelines (Appendix S1).

### 2.1 Setting

The study was conducted in three hospitals in Southeastern Norway. The inclusion of hospitals was pragmatic and based on established collaboration; however, we aimed at including both small and large hospitals. Hospital 1 has a catchment area of 75 000 inhabitants, performing about 200 pediatric surgeries annually. Hospital 2 has a catchment area of 200 000 inhabitants, performing about 380 pediatric surgeries annually. Hospital 3 has a catchment area of 240 000 habitants, performing about 1000 pediatric surgeries annually.

### 2.2 Sample

A purposeful sampling strategy was used, including parents consecutively. Inclusion criteria were parents of children < 18 years of age undergoing surgery in general anesthesia. Surgeries included ear‐nose‐throat, gastric, urologic, and eye. Exclusion criteria were emergencies and situations where parents were not able to understand Norwegian sufficiently to complete the questionnaire.

No sample size calculation was performed. In national surveys of user experiences, a sample of 100–200 respondents from each hospital is assumed sufficient [12]. The data collection was initiated in hospital 3, using the results to psychometrically test and validate the questionnaire. Here, data collection was conducted in August, September, and October 2022, aiming to reach a sample > 100. Data collection in hospitals 1 and 2 were conducted in the 1‐year period September 2023 to September 2024.

Parents were recruited by study nurses at each hospital when the child was ready for discharge from the postoperative anesthesia care unit (PACU). At this time, the child was awake, had eaten and drunk, and had received analgesics. Based on experience, this is where the parents' stress level is lowest.

### 2.3 Data Collection

We used the “pediatric anesthesia parent satisfaction survey (PAPS)” questionnaire [13]. The English version was back translated according to Wild et al. [14], piloted in 10 parents who confirmed the face and content validity, and then psychometrically tested in the responses from Hospital 3 (*n* = 112). The principal component analysis was satisfactory, with a Kaiser‐ Meyer‐Olkin = 0.94 and a significant Bartlett's test (*p* ≤ 001). Spearmans' correlation analysis found significant associations (*p* < 0.01 and *p* < 0.05) between items, varying from 0.2 to 0.9, and the NPAPS was found valid. An article presenting the validation is under review elsewhere. The Norwegian version, NPAPS, comprises 15 statements regarding information and explanation, preoperative experience, postoperative symptom control, integrity, respectfulness and professionality, and overall quality of anesthesia care. Response alternatives were on a 5‐point Likert scale, where 1 = strongly disagree, 2 = disagree, 3 = neither disagree nor agree, 4 = agree, and 5 = strongly agree. Demographic questions included hospital, surgical specialty (ear‐nose‐throat, gastrologic, urologic, eye), age of the child (in years), whether the parent at a previous point of time had followed the current or another child to anesthesia (yes/no), and the relation to the patient (mother/father/other). Also, a final free‐text question allowed for respondents to add anything else about their experience.

After providing their oral consent to participate, parents completed the NPAPS on a tablet, through “Nettskjema.no” a survey solution developed and hosted by the University of Oslo (nettskjema@usit.uio.no).

### 2.4 Analysis

The Statistical Package for the Social Sciences (SPSS) version 28 was used in the analysis. Descriptive statistics and frequencies were used to present the study sample, as well as the responses to each statement. Results are presented as *n* (%), and median (interquartile range, IQR) due to not normally distributed data. The Kruskal–Wallis test was used to assess potential differences between hospitals. A linear regression was used to identify the association between independent variables and overall parent satisfaction. No method for calculating missing data was used. Cronbach's alpha was used to examine the internal consistency of the NPAPS. A *p*‐value < 0.05 was assumed to be statistically significant.

The free‐text responses were assessed following three steps inspired by thematic analysis [15]: (1) familiarization with the data through reading and re‐reading (ACLL), (2) inductive coding, marking all key issues that made sense to the study aims (ACLL), and (3) developing themes across the dataset (ACLL, BTV, AD, CJ, IMB, EDJ).

## 3 Results

In total, 234 parents responded to the questionnaire. A description of the study sample is provided in Table 1.

**TABLE 1:** Study population (*N* = 234).

	Hospital 1 (*n* = 38)	Hospital 2 (*n* = 84)	Hospital 3 (*n* = 112)	Total (*N* = 234)
Age, median (IQR)	8 (5–12)	9 (6–12)	5 (3–10)	7 (4–11)
**Specialty, *n* (%)**				
Ear‐nose‐throat	7 (18.4)	1 (1.2)	80 (72.7)	88 (37.8)
Urologic	16 (42.1)	30 (35.7)	5 (4.5)	51 (21.9)
Gastrologic	2 (5.3)	11 (13.1)	13 (11.8)	26 (11.2)
Eye	—	—	8 (7.4)	8 (3.4)
Unsure	14 (34.2)	42 (50)	4 (3.6)^b^	60 (25.8)
**Experience, *n* (%)**				
Yes	23 (60.5)	38 (45.8)	46 (41.4)	108 (46.4)
No	15 (39.5)	45 (54.2)^a^	65 (58.6)^a^	125 (53.6)
**Relation, *n* (%)**				
Mother	32 (84.2)	59 (70.2)	82 (73.2)	173 (74)
Father	5 (13.2)	25 (29.8)	30 (26.8)	60 (25.5)
Other	1 (2.6)	—	—	1 (0.4)

*Note*: Experience = have you previously ever followed a child to surgery/anesthesia? Relation = relation to the child.

Abbreviation: IQR, interquartile range.

^a^ 1 missing.

^b^ 2 missing.

Table 1 shows that the age of the pediatric patients was a median 7 years (IQR = 4–11). The most frequent surgery was ear‐nose‐throat (37.8%). About half of the respondents had previously followed a child to surgery and anesthesia (46.4%), and “mother” was the most frequent relation to the pediatric patient (74%).

### 3.1 Responses Across Hospitals

Table 2 presents the responses to the NPAPS statements in each hospital. Due to the extensiveness, the response alternative “neither disagree nor agree” is omitted from the table. However, this is visible in the difference between disagree+agree and 100%.

Table 2 shows that nearly all respondents agreed that the anesthesia team answered their questions (94.6%–97.6%), that the information was understandable (96.4%–97.4%), and that the amount of information was appropriate (96.4%–97.4%). Moreover, most respondents agreed that their child's integrity was respected (94.6%–97.4%), that the child was treated respectfully and professionally (96.4%–97.6%) both by other personnel and the anesthesia team, and that they paid attention to the parent's concerns (93.8%–94.7%). Fewer respondents knew who the nurse anesthetist was (84.5%–92.1%) and would recommend the anesthesia team to others (88%–91.9%).

The items with most “disagree” responses were related to being explained how the child might feel physically and emotionally after anesthesia (9.5%–10.5%), and to being satisfied with the way the child fell asleep and woke up from anesthesia (5.3%–7.1%). The items where most respondents were neutral (neither disagree nor agree) were related to pain control postoperatively (7.9%–16.4%) and control of nausea and vomiting (7.9%–22.5%).

Overall, most respondents agreed that their child had received the highest quality care during the surgical experience (96.4%–97.4%).

### 3.2 Differences Between Hospitals

There were few statistically significant differences between hospitals on the NPAPS statements. Fewer agreed that the child's pain was well controlled in Hospital 3 (80%) than in Hospital 2 (84.1%) and Hospital 1 (89.5%) (*p* = 0.04). Also, more respondents in Hospital 1 agreed that the child's nausea and vomiting were well controlled (89.5%) versus in Hospital 2 (73.8%) and Hospital 3 (73.9%) (*p* = 0.04). Regarding recommending the anesthesia team to other family members, 91.9% in Hospital 3 agreed versus 89.5% in Hospital 1 and 88% in Hospital 2 (*p* < 0.01).

### 3.3 Associations

When running a linear regression between the NPAPS statement “I was satisfied with the care my child received from the anesthesia team” and the factors the child's age, surgical specialty, hospital, whether the parent previously had followed a child to surgery and relation to the patient, no statistically significant associations were identified (Table 3).

**TABLE 3:** Regression analysis.

	Beta	Sig.
Hospital	0.39	0.60
Child's age	−0.012	0.88
Surgical specialty	0.045	0.59
Previously followed a child to anesthesia	−0.118	0.09
Relation to the child	0.078	0.24

**TABLE 2:** Responses to the PAPS‐no (*N* = 234).

	Hospital 1 (*n* = 38)		Hospital 2 (*n* = 84)		Hospital 3 (*n* = 112)		*p*
	Disagree, *n* (%)	Agree, *n* (%)	Disagree, *n* (%)	Agree, *n* (%)	Disagree, *n* (%)	Agree, *n* (%)	
The anesthesia team answered all my questions before my child's surgery	1 (2.6)	36 (94.7)	2 (2.4)	82 (97.6)	6 (5.4)	105 (94.6)	0.19
I was satisfied with the amount of information provided by the anesthesia team	1 (2.6)	37 (97.4)	3 (3.6)	81 (96.4)	4 (3.6)	108 (96.4)	0.07
The information given to me by the anesthesia providers was understandable	1 (2.6)	37 (97.4)	3 (3.6)	81 (96.4)	4 (3.6)	106 (94.6)	0.16
I was satisfied with the way my child fell asleep and woke up from anesthesia	2 (5.3)	36 (94.7)	5 (6)	77 (92.8)	8 (7.1)	103 (92)	0.09
The anesthesia team explained to me how my child might feel physically and emotionally after anesthesia and surgery	4 (10.5)	33 (86.8)	8 (9.5)	71 (84.5)	11 (9.8)	91 (81.3)	0.08
I felt my child's pain was well controlled after surgery	1 (2.6)	34 (89.5)	2 (2.4)	69 (84.1)	4 (3.6)	88 (80)	0.04*
I felt my child's nausea and vomiting were well controlled after surgery	1 (2.6)	34 (89.5)	2 (2.5)	59 (73.8)	4 (3.6)	82 (73.9)	0.04*
My child's privacy was respected at all times by the anesthesia team	1 (2.6)	37 (97.4)	2 (2.4)	80 (95.2)	4 (3.6)	106 (94.6)	0.17
The staff we met for my child's surgery behaved professionally and respectfully	1 (2.6)	37 (97.4)	2 (2.4)	82 (97.6)	4 (3.6)	108 (96.4)	0.06
The anesthesia team behaved professionally and respectfully toward my child	1 (2.6)	37 (97.4)	2 (2.4)	82 (97.6)	4 (3.6)	108 (96.4)	0.14
The anesthesia team paid attention to my concerns regarding my child's care	1 (2.6)	36 (94.7)	2 (2.4)	79 (94)	4 (3.6)	105 (93.8)	0.31
I was satisfied with the care my child received from the anesthesia team	1 (2.6)	37 (97.4)	2 (2.4)	80 (96.4)	4 (3.6)	108 (96.4)	0.14
I know who the nurse anesthetist was and his/her role in my child's care	1 (2.6)	35 (92.1)	6 (7.1)	71 (84.5)	6 (5.4)	96 (86.5)	0.05
I would recommend this anesthesia team to others in my family	1 (2.6)	34 (89.5)	2 (2.4)	73 (88)	4 (3.6)	102 (91.9)	< 0.01*
My child received the highest quality care during this surgical experience	1 (2.6)	37 (97.4)	2 (2.4)	81 (96.4)	4 (3.6)	107 (96.4)	0.09

*Note*: Response alternatives strongly disagree and disagree = disagree; agree and strongly agree = agree. The response alternative unsure has not been included, and represent the missing *n*'s. This means that the reported total % above not always adds to 100. Kruskal–Wallis test.

* Statistically significant at a *p* < 0.05‐level.

When controlling for the child's age, surgical specialty or hospital, this did not change. Linear regression between the item “The anesthesia team explained to me how my child might feel physically and emotionally after anesthesia and surgery” and the factors, identified a significant association with “relation to the child” (Beta = 0.177, *p* < 0.01). Beyond this, no other significant associations between any of the NPAPS items and the factors were identified, even if controlling for the child's age, surgical specialty, or hospital.

### 3.4 Free‐Text Responses

In total, 53 respondents added a free‐text response. Of these, 50 only had positive comments on their experience. These included a calm and safe setting, with personnel exhibiting trust and using diversion techniques and humor to comfort the child. Three respondents added negative comments, including long waiting times (*n* = 2), little information during waiting time (*n* = 1), and little information regarding where to attend (*n* = 1).

## 4 Discussion

This is the first study exploring parent satisfaction with pediatric anesthesia in Norway. The results indicate that even if most parents were satisfied with the information provided, and with being treated professionally and with respect, there were also areas that can be improved. These include better explanations about postoperative symptoms and postoperative symptom control. Hospital, type of surgery, the child's age, whether the parent previously had followed a child to anesthesia, and the parents' relation to the child did not seem to impact the overall satisfaction with anesthesia care.

In this study, parents were overall satisfied with the anesthesia care for their child. This aligns with previous studies indicating high parent satisfaction (> 90%) [10, 13, 16, 17]. Yet, it also contrasts with a study from Ethiopia, where only 77.7% reported being satisfied [8]. This may be due to anesthesia services not being so developed and/or available in low‐income countries, facing challenges such as a shortage of skilled personnel, inadequate resources, and limited training opportunities [18].

Moreover, our results indicate improvement areas regarding explaining how the child might feel physically and emotionally after anesthesia, the way the child potentially will fall asleep and wake up from anesthesia, and regarding postoperative control of pain, nausea, and vomiting. This aligns with the findings of Milliken‐Glabe et al. [13]. Also, in our study, three respondents reported negatively on waiting times. We have not identified studies exploring the impact of waiting times in an anesthesia context. However, studies from emergency departments indicate that waiting times are not associated with satisfaction in pediatric contexts [19], yet they are seen as a timely quality indicator [20].

In this study, we did not see any associations between parent satisfaction and factors such as the child's age, surgical specialty, whether the parent previously had followed a child to surgery, and relation to the patient. Previous studies have shown associations between satisfaction and parents' or children's level of anxiety preoperatively [8, 17], or the administration of sedative premedication [8]. As such, a systematic review and meta‐analysis found that parental presence at induction of anesthesia reduces parental and patient anxiety, may increase parental satisfaction, and may not impact operating room efficiency [21]. However, whether parents' presence is useful from anesthesia personnel's perspective relies on their ability to both support the child and shelter their own feelings during the process [22]. Also, parents experiences with being present vary from being positive to being traumatic [23] As such, parental presence remains controversial and has been reported to provide mixed results [24, 25]. In Norway, parents are most commonly present at induction of anesthesia, as was the case in the current hospitals. This may have impacted on the NPAPS scoring.

Even if we found few statistically significant differences between hospitals to the NPAPS statements, there were some differences regarding pain or nausea and vomiting control. This finding is not necessarily interesting to a broader audience. However, it underlines the importance of thorough planning of the pediatric anesthesia pathway.

The limited evidence‐base underlines the need for further exploration of the quality of pediatric anesthesia care, both through parent or patient satisfaction, but also through assessment of adverse outcomes such as pain, nausea, vomiting, possibly leading to further adverse outcomes [3, 4]. A 2022 systematic review and meta‐analysis [26] suggests that family‐centered educational interventions may be beneficial to reduce pediatric and parental anxiety and improve pediatric behaviors at induction of anesthesia. However, its efficacy regarding postoperative behaviors and/or symptom presence is undiscovered. As such, a 2024 narrative review about the use of preoperative time underscores the importance of anesthesia provider skills, combined with a careful pre‐anesthetic mindset toward patients and their families that “recognizes the interwoven relationship between children and parents.” The authors conclude that standardized protocols and guidelines in pediatric anesthesia are needed to enhance clinical efficacy [27]. Also, this study supports the development of tools that measure quality of recovery more broadly in children, like there is in adult perioperative medicine [28]. The Pediatric Perioperative Outcomes Group has been established to take up the task of pursuing the question “How do we measure/define a successful anesthetic in infants, children, and young people?” PEVuZE5vdGU [29].

The value of measuring parent satisfaction as a quality indicator may be discussed. The terms «satisfaction», «preferences», and «experiences» have been used interchangeably, despite their different meanings [30]. Satisfaction can be defined as the gap between respondents' expectations and experiences. Hence, patient‐reported experiences may be less subjective than reported satisfaction [7, 31]. As stated by Larsson et al. [32] these issues should be addressed during the design phase to increase the usability of the study measures.

### 4.1 Strengths and Limitations

A strength of this study is the inclusion of respondents from three hospitals of various sizes. However, a sample size calculation was not performed, and the sample is limited to one country, respondents in children aged 3–12 years, and in a limited selection of elective surgeries. We did not register parents declining to participate, and there is a potential selection bias. However, the feedback from study nurses was that most parents fulfilling the inclusion criteria agreed to participate. Moreover, we cannot calculate the response rate. In Hospitals 1 + 2, the number of respondents accounts for approximately 20% of the annual patient population. If evenly distributed throughout the year, Hospital 3 has about 250 patients in 3 months, and an assumed response rate here was 45%. Of course, this limits the generalizability of our findings. Still, the results were coinciding across the three hospitals, and this strengthens the external validity of our findings. Also, our results add to the limited evidence‐base internationally.

Further, the clinical significance of our results may be discussed. As experienced clinicians and researchers, we suggest that hospitals use the results as an indication of areas that may need improvement to aim for optimal parental satisfaction and quality anesthesia care.

Another strength of this study is the use of a validated and psychometrically tested questionnaire. Also, the Cronbach's alpha was 0.96, showing a very high internal consistency of the questionnaire. However, the quantitative design only allows for responding to the determined questions, not opening for in‐depth exploration. Also, the free‐text responses were limited and keyword‐based, not allowing for further thematic analysis.

Even if parent satisfaction is seen as a proxy for children's satisfaction, for example, [33] found conflicting results when comparing parents' and children's perioperative experiences. This underlines the need for further research.

## 5 Conclusion

Even if parents are overall satisfied with anesthesia care and information, and being treated professionally and with respect, some areas should be focused. Preoperative education of parents could improve their preparedness regarding behavior and symptoms during induction and emergence of anesthesia. Also, “anesthesia personnel” awareness of pain, nausea, and vomiting control must never rest. Further studies should explore interventions that increase parent and patient satisfaction, as well as the overall quality of pediatric anesthesia care.

## Author Contributions

A.C.L.L., B.T.V., A.D., C.J., I.M.B., and E.D.J. have all made substantial contributions to the conception and design of the study. A.D., C.J., I.M.B., and E.D.J. contributed to the acquisition of data. A.C.L.L. was responsible for the analyses and interpretation of data and wrote the initial draft of the manuscript. All authors (B.T.V., A.D., C.J., I.M.B., and E.D.J.) were involved in revising it critically for important intellectual content. All authors have given final approval of the version to be published and agreed to be accountable for all aspects of the work in ensuring that questions related to the accuracy or integrity of any part of the work are appropriately investigated and resolved.

## Ethics Statement

The study was based on guidelines for ethical research. A willing, informed consent to participate was obtained from all participants. The study was assessed by the Regional Committees for Medical and Health Research Ethics (REC) in Norway, which stated that no approval was needed (reference no. 456756). The study was assessed and approved by the data protection officer at each of the participating hospitals.

## Consent

The authors have nothing to report.

## Conflicts of Interest

The authors declare no conflicts of interest.

## Supporting information


Appendix S1.

## Data Availability

The datasets used and/or analyzed are available from the corresponding author upon reasonable request.
